# CoCoNat: a novel method based on deep learning for coiled-coil prediction

**DOI:** 10.1093/bioinformatics/btad495

**Published:** 2023-08-04

**Authors:** Giovanni Madeo, Castrense Savojardo, Matteo Manfredi, Pier Luigi Martelli, Rita Casadio

**Affiliations:** Biocomputing Group, Department of Pharmacy and Biotechnology, University of Bologna, Italy; Biocomputing Group, Department of Pharmacy and Biotechnology, University of Bologna, Italy; Biocomputing Group, Department of Pharmacy and Biotechnology, University of Bologna, Italy; Biocomputing Group, Department of Pharmacy and Biotechnology, University of Bologna, Italy; Biocomputing Group, Department of Pharmacy and Biotechnology, University of Bologna, Italy

## Abstract

**Motivation:**

Coiled-coil domains (CCD) are widespread in all organisms and perform several crucial functions. Given their relevance, the computational detection of CCD is very important for protein functional annotation. State-of-the-art prediction methods include the precise identification of CCD boundaries, the annotation of the typical heptad repeat pattern along the coiled-coil helices as well as the prediction of the oligomerization state.

**Results:**

In this article, we describe CoCoNat, a novel method for predicting coiled-coil helix boundaries, residue-level register annotation, and oligomerization state. Our method encodes sequences with the combination of two state-of-the-art protein language models and implements a three-step deep learning procedure concatenated with a Grammatical-Restrained Hidden Conditional Random Field for CCD identification and refinement. A final neural network predicts the oligomerization state. When tested on a blind test set routinely adopted, CoCoNat obtains a performance superior to the current state-of-the-art both for residue-level and segment-level CCD. CoCoNat significantly outperforms the most recent state-of-the-art methods on register annotation and prediction of oligomerization states.

**Availability and implementation:**

CoCoNat web server is available at https://coconat.biocomp.unibo.it. Standalone version is available on GitHub at https://github.com/BolognaBiocomp/coconat.

## 1 Introduction

Coiled-coil domains (CCD) in proteins are structural motifs where α‐helices pack together in an arrangement called knobs into holes (Crick 1952, 1953a, b). Since the first crystallographic observation in the structure of influenza virus hemagglutinin (Wilson *et al.* 1981), CCDs have been resolved in several proteins through all the kingdoms of life (Truebestein and Leonard 2016). CCDs are present, among others, in structural proteins, transcription factors, and enzymes (Truebestein and Leonard 2016, Lupas and Bassler 2017). CCDs act as molecular spacers, influence the organelle organization, constrain the distance of residues involved in binding and catalytic sites, mediate membrane fusion, and are involved in signal transduction and solute transport.

Canonical CCDs include the interaction of two or more α‐helices, wound around each other and forming a supercoiled bundle. Each helix is characterized by the repetition of a seven-residue motif (heptad repeat) whose positions are referred to as registers and are labeled as *abcdefg*. Positions *a* and *d* are routinely occupied by hydrophobic residues and mediate the interaction between different helices in the domain. As a result of the formation of the supercoil bundle, the effective periodicity of α‐helices in CCDs changes from 3.6 to 3.5 residues per turn (Lupas and Gruber 2005, Lupas *et al.* 2017, Szczepaniak *et al.* 2020). This implies that residues in the same register lie on the same side of the helix surface. Therefore, the hydrophobic nature of residues in registers *a* and *d* confers a peculiar amphipathic character to the α‐helix. In CCDs, α‐helices interact with each other through their hydrophobic face (Lupas and Gruber 2005). CCDs can contain helices characterized by non-canonical repeats, longer than seven residues, i.e. hendecades, pentadecades, and nonadecades (Lupas and Gruber 2005, Szczepaniak *et al.* 2020). This article does not take into consideration these cases.

CCDs are classified according to the number and orientation of the involved α‐helices, i.e. by their oligomerization state. CCDs based on the orientation of helices are classified as parallel or antiparallel and based on the number of helices as dimers, trimers, and tetramers.

CCDs are routinely annotated starting from the protein 3D structure, adopting specialized software such as SOCKET (Walshaw and Woolfson 2001) and SamCC-Turbo (Szczepaniak *et al.* 2020). Annotations performed with the two methods are collected in the CC+ database (http://coiledcoils.chm.bris.ac.uk/ccplus/search/, Testa *et al.* 2009) and in the CCdb database (https://lbs.cent.uw.edu.pl/ccdb), respectively. Semi-manual annotations are also available in the SCOPe database (Fox *et al.* 2014).

The relevance of CCDs in protein annotation requires the development of computational methods for predicting the presence and localization of CCDs (including registers), and their oligomerization state, starting from the protein sequence. Over the years, several methods have been proposed, addressing the different tasks of CCD prediction. Boundaries of α-helices involved in CCDs can be predicted with COILS (Lupas *et al.* 1991), PCOILS (Gruber *et al.* 2005), MARCOIL (Delorenzi and Speed 2002), Multicoil2 (Trigg *et al.* 2011), CCHMM_PROF (Bartoli *et al.* 2009), DeepCoil (Ludwiczak *et al.* 2019), and CoCoPRED (Feng *et al.* 2022).The oligomeric state is predicted with PrOCoil (Mahrenholz *et al.* 2011), LOGICOIL (Vincent *et al.* 2013), and CoCoPRED (Feng *et al.* 2022). To date, methods predicting the registers along the heptad are MARCOIL (Delorenzi and Speed 2002), Multicoil2 (Trigg *et al.* 2011), DeepCoil2 (Ludwiczak *et al.* 2019), and CoCoPRED (Feng *et al.* 2022).

Recently protein language models improved sequence encoding procedures. Here we introduce CoCoNat which, for the first time, adopts a sequence encoding based on the combination of two state-of-the-art protein language models, ProtT5 (Elnaggar *et al.* 2021) and ESM2 (Lin *et al.* 2023) and based on deep-learning computes: (i) the coiled-coil helix boundaries; (ii) the residue-level register annotation, and (iii) the CCD oligomerization state.

We trained CoCoNat on a dataset comprising 2198 proteins containing CCDs and 9062 proteins without CCD (negative examples). When tested on a blind test set including 400 CCD and 318 non-CCD proteins, CoCoNat scores with a performance that is superior to the current state-of-the-art both for residue-level and segment-level CCD detection. Moreover, CoCoNat significantly outperforms other methods, on both register annotation and prediction of CCD oligomerization state.

## 2 Materials and methods

### 2.1 Datasets

CoCoNat is trained and tested on the same datasets adopted in CoCoPRED (Feng *et al.* 2022). Numbers are summarized in Table 1 and all datasets are available at the CoCoNat website (https://coconat.biocomp.unibo.it). Additional statistics on the oligomeric state classification of coiled-coil helices in the training and testing sets are reported in Supplementary Fig. 1.

**Table 1.** Training and testing set of CoCoNat.

	Positive proteins	Helices in CCDs	Negative proteins
Training set	2191	4342	9040
Blind test set	400	863	318

#### 2.1.1 Training dataset

The positive training dataset contains 2191 proteins out of the 2337 included in CoCoPRED and deriving from 30,227 CCD-containing proteins annotated with SOCKET (Walshaw and Woolfson 2001) in the CC+ database (Testa *et al.* 2009).

Briefly, proteins included in the positive training set of CoCoPRED have been selected with the following criteria: (i) protein structure resolution < 4 Å, (ii) protein length between 25 and 700 residues, (iii) length of CCD helices ≥ 8 residues; (iv) absence of non-canonical (i.e. non-heptad based) CCDs; (v) CCD oligomeric state classified as parallel or antiparallel dimer, trimer, and tetramer; (vi) sequence pairwise identity lower than 30% with respect to proteins in the testing set (see below); and (vii) internal pairwise sequence identity lower than 50%.

Since CoCoNat relies on full length proteins for the computation of sequence embeddings, we applied further filters to the CoCoPRED positive training dataset, removing: (i) proteins not mapped into UniProt; (ii) synthetic and fusion proteins; and (iii) proteins whose structure coverage with respect to the UniProt sequence is lower than 70%. After this screening, the positive dataset includes 2191 proteins with 4342 coiled-coil helices, whose length ranges from 8 to 145 residues. The number of coiled-coil helices per protein ranges from 1 to 19.

The negative training set of CoCoNat includes 9040 proteins. This derives from the 9358 proteins of the negative set of CoCoPRED that was obtained from the negative set of DeepCoil (Ludwiczak *et al.* 2019) after the exclusion of proteins with a sequence identity > 30% with respect to the blind test set (see Section 2.1.2) and with a sequence identity > 50% with respect to the positive training set. We filtered out proteins not mapped into UniProt.

Proteins in the training set (both positive and negative examples) were split into 10 subsets for 10-fold cross-validation. To reduce the redundancy among cross-validation sets, proteins sharing more than 25% sequence identity at 50% coverage are clustered in the same set. Cross-validation sets were used to set all the hyperparameters.

#### 2.1.2 Blind test dataset

To test and benchmark CoCoNat with other available methods, we adopted the 718 proteins included in the CoCoPRED test set. The CoCoPRED set shares less than 30% sequence identity with proteins in the training sets of CCHMM_PROF (Bartoli *et al.* 2009), MARCOIL (Delorenzi and Speed 2002), Multicoil2 (Trigg *et al.* 2011), CoCoPRED (Feng *et al.* 2022), and DeepCoil2 (Ludwiczak *et al.* 2019).

Since coiled-coil annotation for this dataset was not available from the CoCoPRED website, we ran SOCKET in house on the structure of the main biological assembly of each PDB included in the dataset. By this, 400 proteins are annotated as containing CCD while 318 do not contain any coiled-coil segment. Overall, the 400 positive proteins contain 863 coiled-coil helices (Table 1).

### 2.2 Protein encoding

CoCoNat makes use of residue embeddings obtained with large-scale protein language models (pLMs) to represent proteins in training and testing sets. Specifically, we adopted two state-of-the-art pLMs: ProtT5 (Elnaggar *et al.* 2021) and ESM2 (Lin *et al.* 2023), generating, for each residue in the protein sequence, 1024 and 1280 features, respectively. The ESM2 model has been released in several versions, based on different transformer architectures with a varying number of cascading layers. In order to limit the resources required to compute the representations and having an embedding dimension comparable to that provided by ProtT5, we adopted the intermediate model, providing representations of 1280 components and comprising 33 transformer layers with 650M of parameters. Residue-level representations are then concatenated together, leading to vectors of 2304 dimensions for each residue in the sequences. The concatenation of embeddings obtained with different pLMs has been shown to improve the performance in previous works (Manfredi *et al.* 2022, 2023).

### 2.3 CoCoNat architecture

**Figure 1.**
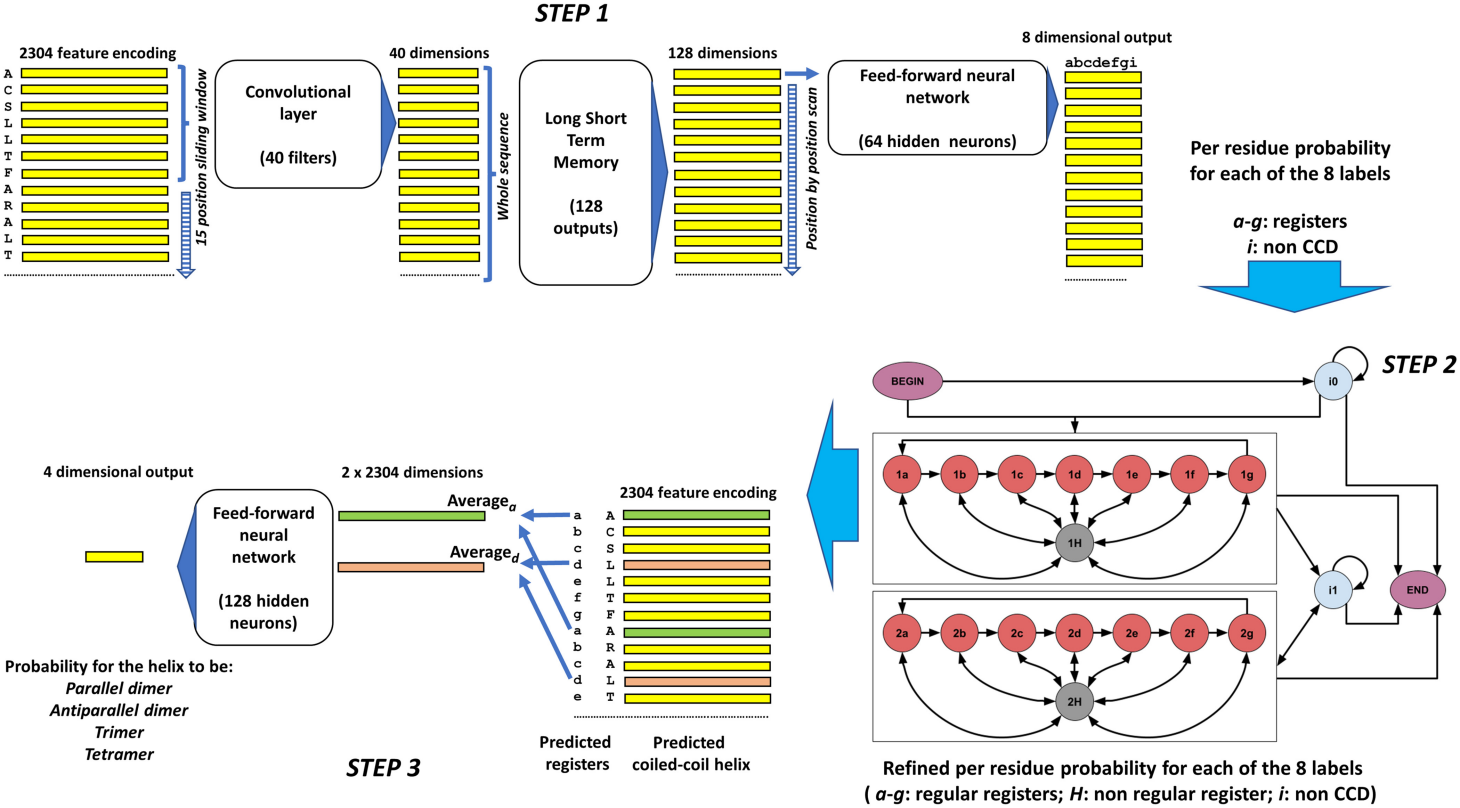
Workflow of the predictive model of CoCoNat, comprising three steps. The *step 1* maps each residue, encoded with a 2304-dimensional vector (the concatenation of ProtT5 and ESM2 embeddings) into an eight-dimensional vector representing the probability distribution over the labels (*a–g* registers for coiled-coil helices and *i* for non-CCD portions). It combines in cascade (i) a convolutional layer that computes a 40-dimensional representation for each position in the sequence, based on a sliding window of 15 contiguous residues, (ii) an LSTM layer that analyzes the whole sequence and provides a 128-dimensional representation for each position, and (iii) a fully connected feed-forward NN that, residue by residue, provides the 8-dimensional mapping. The *step 2* consists of a GRHCRF that casts the grammar of CCDs in the topology of the connections among 20 different states. Each sequence is generated by a path that starts from the BEGIN state and can either enter the self-looping *i*0 state, which models the *N*-terminal non-CCD portion of the protein, or the first eight-state block (*1a–1b–1c–1d–1e–1f–1g–1H*), which models the first CCD domain. Labels *a–g* of the states in the block correspond to registers and state *H* accommodates non-regular transitions. Residues after the first coiled-coil helix are modeled by the *i*1 state (non-CCD) and, in case, by a second CCD block (*2a–2b–2c–2d–2e–2f–2g–2H*), analogous to the first one. All states, but *1H* and *2H*, can make transition to the END state, terminating the path. GRHCRF provides the annotation of coiled-coil helix boundaries and of registers by computing the optimal *a posteriori* Viterbi path, given the probabilities computed in the step 1. The *step 3* provides the prediction of the oligomeric state, based on the annotation computed in step 2 and on the 2304-dimensional embedding. For each predicted coiled-coil helix, embeddings labeled with registers *a* and *d* are separately averaged and fed into a feed-forward NN that computes the probability distribution over the four possible classes (parallel and antiparallel dimer, trimer, and tetramer). The three steps of the architecture are trained separately.

CoCoNat is organized as a three-step method combining a deep learning approach, a probabilistic graphical model, and a single-layer neural network (NN) in a cascading way. The three different steps of the model are trained independently from each other. The first two steps are collectively devised for detecting coiled-coil helix boundaries and the residue-level annotation of registers within each predicted helix. The third step predicts the CCD oligomerization state (Fig. 1). In the following sections, each step is briefly described.

#### 2.3.1 Deep learning architecture

The first step (Fig. 1) is based on a convolutional layer (LeCun *et al.* 1989) followed by a long short-term memory (LSTM) layer (Hochreiter and Schmidhuber 1997). The convolutional layer captures local dependencies of the input data. This layer, adopting a 15 residue long sliding window, takes as input the protein, where each residue is represented with a 2304-feature vector. By applying 40 different filters, the layer outputs the same protein with residues encoded with 40-feature vectors. This mapping is provided as input to a LSTM layer including 128 output neurons, which captures long-range dependencies. Finally, we apply a standard feed-forward network (with 64 hidden neurons) with 8 output neurons (one for each possible coiled-coil registers, *a*–*b–c–d–e–f–g*, plus one, *i,* for non-CCD residues), endowed with a sigmoid activation function. The output gives the per-residue probability of each register or none.

In order to reduce overfitting, we introduce dropout layers between convolutional, LSTM, and the feed-forward network with rate fixed to 0.25. The architecture was trained with a gradient descent of the Kullback–Leibler divergence error function with the Adam optimization algorithm (Kingma and Ba 2017). The Kullback–Leibler loss was chosen since it well-fits with models providing probability distributions in output, as in our case. The best model was determined using the early stopping technique of 10 epochs in which the validation error did not decrease.

#### 2.3.2 Refining the prediction with Grammatical-Restrained Hidden Conditional Random Field (second step)

The second step (Fig. 1) takes in input the probabilites computed by step 1. Grammatical-Restrained Hidden Conditional Random Field (GRHCRF) is a discriminative probabilistic model (Fariselli *et al.* 2009, Madeo *et al.* 2021), and it allows to introduce the regular grammar of the CDD registers. In the prediction phase, a posterior-Viterbi dynamic-programming algorithm computes the most probable path along the model, satisfying the grammatical constraints.

In our model, the grammar has two identical blocks for CCD prediction, two states (*i*0 and *i*1 in Fig. 1, step 2) with a self-loop modeling the non-CCD regions, one BEGIN and one END states. In each CCD block, seven states model the register sequence, and one further state (H, 1, and 2) accommodates non-regular transitions (i.e. transitions escaping the regular heptad repeat pattern, *abcdefg*). The first GRHCRF block models the first coiled-coil helix and the second one models all the others coiled-coil helices, when present, in the protein sequence.

The GRHCRF output defines the precise identification of CCD boundaries and annotates the typical heptad repeat pattern along the coiled-coil helices.

#### 2.3.3 Prediction of the oligomerization state

The prediction of the CCD oligomerization state adopts a simple feed-forward NN with a single hidden layer, comprising 128 neurons, and four output units corresponding to the four possible oligomerization states: parallel and antiparallel dimers, trimers, and tetramers.

The input of this network is built based on a well-known biophysical feature: the oligomeric state of canonical CCD is largely determined by the nature of hydrophobic residues in the heptad repeat pattern, namely, residues labeled with registers *a* and *d* (Woolfson 2023; Li *et al.* 2016). Based on this observation, for a given coiled-coil helix, the network input is obtained concatenating the average embedding vectors of *a* and *d* predicted positions.

More formally, given a coiled-coil helix of length l, with an embedding matrix *E* of dimension l×2304 (as derived from the concatenation of two pLM embeddings, ProtT5 and ESM2), the following two mean vectors are computed: where *N_a_* and *N_d_* are the number of positions labeled with registers *a* and *d*, respectively. The input vector for the network is then obtained concatenating ${\bar{e}}_{a}$ and ${\bar{e}}_{d}$: where $\left[\cdot \right]$ denotes the vector concatenation operator.


(1) $${\bar{e}}_{a}=\frac{1}{N_{a}}\sum_{r=a}E_{r}$$

(2) $${\bar{e}}_{d}=\frac{1}{N_{d}}\sum_{r=d}E_{r}$$

(3) $$x={\bar{e}}_{a}\cdot {\bar{e}}_{d}$$

#### 2.3.4 Model training and selection procedure

The whole CoCoNat architecture was trained with three independent training procedures for steps 1, 2, and 3.

The deep-learning architecture of step 1 was optimized using 10-fold cross-validation and a grid search to select the main model hyperparameters. Specifically, we selected the best number of convolutional filters (testing values in the set 10, 20, 40, 80, 160), the optimal LSTM hidden output size (testing values in the set 32, 64, 128, 256), and the optimal number of hidden neurons in the final feed-forward network (testing values in the set 12, 32, 64, 128, 256). The optimal configuration was chosen as the one maximizing the cross-validation *F*1 score at residue level (see next section), and include 40 convolutional filters, 128 units for the LSTM size and 64 neurons in the hidden layer of the final feed-forward network.

For step 2, the hyperparameters of the GRHCRF include the σ^2^ used for L2 regularization and the number of training iterations (Fariselli *et al.* 2009). These were optimized in cross-validation and grid search testing various combinations (σ^2^ in the set 0.0001, 0.001, 0.05, 0.1 and iterations in the set 10, 20, 40, 100). The optimal values are σ^2^= 0.05 and 40 iterations.

Finally, step 3 architecture only required to optimize the number of hidden neurons. Again, we tested different values (16, 32, 64, 128, 256) and selected the optimal one as those maximizing the average MCC across the four oligomeric states. The best value is 128.

Steps 1 and 3 architectures are implemented in PyTorch (https://pytorch.org/). The GRHCRF is implemented using the biocrf package (Fariselli *et al.* 2009).

### 2.4 Scoring performance

To evaluate the performance of our method in recognizing coiled-coil helices, we adopted residue- and segment-based measures.

The residue-based scores include precision (PRE_R_), recall (REC_R_), and *F*1-score (*F*1_R_). where TP_R_, FP_R_, and FN_R_ are true positive, false positive, and false negative coiled-coiled residues, respectively.


(4) $${\mathrm{PRE}}_{R}=\frac{{\mathrm{TP}}_{R}}{{\mathrm{TP}}_{R}+{\mathrm{FP}}_{R}}$$

(5) $${\mathrm{REC}}_{R}=\frac{{\mathrm{TP}}_{R}}{{\mathrm{TP}}_{R}+{\mathrm{FN}}_{R}}$$

(6) $${F1}_{R}=\frac{2\times {\mathrm{PRE}}_{R}\times {\mathrm{REC}}_{R}}{{\mathrm{PRE}}_{R}+{\mathrm{REC}}_{R}}$$

Analogously, the segment-based scores include precision (PRE_S_), recall (REC_S_), and *F*1-score (*F*1_S_):


(7) $${\mathrm{PRE}}_{S}=\frac{{\mathrm{TP}}_{S}}{{\mathrm{TP}}_{S}+{\mathrm{FP}}_{S}}$$

(8) $${\mathrm{REC}}_{S}=\frac{{\mathrm{TP}}_{S}}{{\mathrm{TP}}_{S}+{\mathrm{FN}}_{S}}$$

(9) $${F1}_{S}=\frac{2\times {\mathrm{PRE}}_{S}\times {\mathrm{REC}}_{S}}{{\mathrm{PRE}}_{S}+{\mathrm{REC}}_{S}}$$

In this case, TP_S_, FP_S,_ and FN_S_ are computed for the coiled-coil helices. Prediction is considered correct (TP_S_) only if the overlap between predicted and observed segments is at least equal to the half-length of the longest segment.

Following Feng *et al.* (2022), we used two segment overlap (SOV) measures, one taking as reference observed residues (SOVo) and one taking as reference predicted residues (SOVp) (Zemla *et al.* 1999).

We computed the precision-recall (PR) curve and the relative area under the curve (PR-AUC), by plotting the two measures at varying thresholds of coiled-coil probabilities as obtained from the GRHCRF posterior probability values.

For the register and oligomeric state prediction tasks, we reported class-level MCC values:


(10) $$\mathrm{MCC}=\frac{\mathrm{TP}\times \mathrm{TN}-\mathrm{FP}\times \mathrm{FN}}{\sqrt{\left(\mathrm{TP}+\mathrm{FP})(\mathrm{TP}+\mathrm{FN})(\mathrm{TN}+\mathrm{FP})(\mathrm{TN}+\mathrm{FN}\right)}}$$

## 3 Results

### 3.1 Visualizing ProtT5 and ESM2 embeddings with t-SNE

For evaluating whether the two language models capture coiled-coil-related features in their representations, we adopted *t*-SNE (van der Maaten and Hinton 2008) to project raw embedding vectors obtained with ProtT5 and ESM2 in two dimensions.

First, we wanted to assess if the raw embeddings can distinguish the different register features. To this aim, we projected all representations of coiled-coil residues in the training set, and then we colored projected points by the heptad repeat register they are annotated with, distinguishing two classes: hydrophobic register positions (*a* and *d*) and polar positions (*b*, *c*, *e*, *f*, and *g*). Results are shown in Supplementary Fig. 2A. From the projections, even if a clear separation is missing, it is evident that the two models can capture the hydrophobic/polar register difference to a good extent. Among the two models, ProtT5 seems to provide a slightly better separation.

Second, to investigate if the embedding can capture the relation between hydrophobic registers and oligomeric state, we projected all representations of hydrophobic register positions in the training set (*a* and *d*), and then we colored projected points according to the oligomeric state of the corresponding helix. Resulting *t*-SNE projections are shown in Supplementary Fig. 2B. In this case, the separation is less evident, even if some clusters are visible (mostly in ProtT5 projections) for parallel dimers and trimers. Possibly, more *t*-SNE dimensions are needed to achieve a better separation.

Overall, these experiments suggest that information is already present in the raw embeddings. However, a specific transfer learning architecture, processing the whole sequence, and further exploiting the local and global sequence context is needed to better capture coiled-coil features.

### 3.2 Cross-validation results

We performed 10-fold cross-validation experiments to evaluate the contribution from the two protein language models. We independently trained three different identical models adopting as input: (i) ProtT5, (ii) ESM2, and (iii) both encodings combined in a single vector. Results are reported in Table 2. The overall prediction achieved with each one of the two language models is similar (*F*1_R_ and *F*1_S_ scores equals 0.44, 0.34, and 0.46, 0.37 with ProtT5 and ESM2, respectively). However, combining the two embeddings into a single vector leads to better performances, raising both precision and recall values, and achieving *F*1_R_ and *F*1_S_ values of 0.52 and 0.41, respectively. This suggests that the two models are somewhat complementary. We analyzed and compared residue-level true positive predictions obtained with inputs based on the two pLMs. We find that the two models individually trained with ProtT5 and ESM2 share 25 373 correctly predicted residues, and that 4166 and 8341 coiled-coil residues are correctly and uniquely identified, respectively. This finding highlights the complementarity of the two models. These results agree with previous works in which the combination of embeddings from different pLMs has been proven effective also for other prediction tasks (Manfredi *et al.* 2022, 2023). All results presented in this manuscript are obtained using the combination of the two input embeddings.

**Table 2.** Prediction of coiled-coil helices with CoCoNat adopting different embeddings.^a^

Input	PRE_R_	REC_R_	*F*1_R_	PRAUC	PRE_S_	REC_S_	*F*1_S_
ProtT5	0.42	0.46	0.44	0.38	0.32	0.36	0.34
ESM2	0.50	0.50	0.46	0.41	0.47	0.32	0.37
ProtT5+ESM2	0.51	0.47	0.49	0.47	0.38	0.37	0.37

a CoCoNat architecture is depicted in Fig. 1. The training set is described in Table 1. Results are obtained adopting a 10-fold cross-validation. For details, see Section 2. Subscript R: per residue; subscript S: per CC helix segment. Variability across cross-validation sets is lower than 1% for all scores.

### 3.3 Prediction of coiled coils on the blind test set

CoCoNat is benchmarked on the same blind test set against available methods. In Table 3, we report results for the prediction of coiled-coil helices. Tested methods include: MARCOIL (Delorenzi and Speed 2002), CCHMM_prof (Bartoli *et al.* 2009), Multicoil2 (Trigg *et al.* 2011), DeepCoil2 (Ludwiczak *et al.* 2019), and CoCoPRED (Feng *et al.* 2022). All the methods were run in house using the respective available standalone versions. Since DeepCoil2 does not provide a classification but rather a probability value, we report results obtained by applying two different probability thresholds set to 0.2 and 0.5, respectively.

**Table 3.** CoCoNat and the state-of-art methods on the same blind test set.

**Method** ^a^	PRE_R_	REC_R_	*F*1_R_	PRAUC	PRE_S_	REC_S_	*F*1s	SOV_O_	SOV_P_
MARCOIL	0.34	0.26	0.29	0.16	0.24	0.06	0.1	18.15	48.03
CCHMM_prof	0.16	**0.6**	0.23		0.12	0.25	0.15	43.25	16.32
Multicoil2	0.34	0.13	0.19	0.15	0.19	0.01	0.02	7.40	48.86
DeepCoil2 (th = 0.2)	0.39	**0.6**	0.48	0.36	0.42	**0.49**	0.45	**59.83**	50.93
DeepCoil2 (th = 0.5)	0.51	0.33	0.4	0.36	0.53	0.2	0.29	31.17	66.64
CoCoPRED	0.43	0.54	0.48	0.45	0.38	0.46	0.41	57.64	51.17
CoCoNat	**0.55**	0.53	**0.54**	**0.46**	**0.57**	0.43	**0.49**	54.35	**66.93**

a MARCOIL (Delorenzi and Speed 2002), CCHMM_prof (Bartoli *et al.* 2009), Multicoil2 (Trigg *et al.* 2011), DeepCoil2 (Ludwiczak *et al.* 2019), CoCoPRED (Feng *et al.* 2022). Subscript R: per residue; subscript S: per CC helix segment. The blind test set contains 400 positive and 318 negative proteins. Bold values highlight the highest scores.

CoCoNat, which adopts encodings based on ProT5 and ESM2, outperforms the state-of-the-art, showing (with respect to the second top performing method in the benchmark) an improvement in the per-residue precision value (0.55), with a slight loss in recall (0.53), which is reflected in the higher value of the *F*1-score (0.54). The per-segment scores of CoCoNat confirm this trend. Moreover, CoCoNat performs with the highest SOVp and the third highest SOVo (see Section 2.4 for definition).

For sake of assessing the significance of the differences observed in data reported in Table 3, we performed a boostrapping procedure and a two-sample Welch’s *t*-test. Specifically, from the blind test set results, we randomly selected 100 samples of 300 sequences and evaluated all the methods using residue- and segment-level scoring measures. Then, average performances of CoCoNat were compared for statistical significance with average scores of other tools. All the differences observed in this experiment reflect those reported in Table 3 and are all significant at 0.0001 significance threshold. Results are reported in Supplementary Table 1.

### 3.4 Prediction of coiled-coil registers

We compared CoCoNat with other tools in the task of annotating heptad repeat registers. To this aim, we used the blind test of 400 proteins endowed with CCDs. Results of all methods were generated using the respective standalone versions (Table 4).

**Table 4.** CoCoNat and the state-of-art methods on the prediction of heptad repeat registers.

**Method** ^a^	MCC (a)	MCC (b)	MCC (c)	MCC (d)	MCC (e)	MCC (f)	MCC (g)
MARCOIL	0.67	0.67	0.67	0.67	0.66	0.66	0.66
Multicoil2	0.56	0.56	0.57	0.57	0.58	0.58	0.57
DeepCoil2 (th = 0.2)	0.62			0.62			
DeepCoil2 (th = 0.5)	0.68			0.68			
CoCoPRED	0.65	0.67	0.67	0.66	0.66	0.67	0.65
CoCoNat	**0.84**	**0.84**	**0.84**	**0.84**	**0.83**	**0.83**	**0.83**

a MARCOIL (Delorenzi and Speed 2002); Multicoil2 (Trigg *et al.* 2011); DeepCoil2 (Ludwiczak *et al.* 2019); CoCoPRED (Feng *et al.* 2022). The blind test set contains 400 positive proteins. Bold values highlight the highest scores.

For all the register labels *a–g*, CoCoNat MCC values indicate an improvement ranging from 16% to 19% with respect to the second best-performing method, CoCoPRED.

Remarkably, CoCoNat MCC values are quite similar across all the seven different register labels, suggesting that the register is routinely predicted in the correct regular configuration, from label *a* to *g*. This highlights that the GRHCRF grammar is properly capturing transition constraints among the different registers within the coiled-coil segments.

### 3.5 Prediction of the CCD oligomerization state

We finally compared CoCoNat, LOGICOIL (Vincent *et al.* 2013) CoCoPRED (Feng *et al.* 2022) on the task of predicting CCD oligomerization state. Again, we used the blind test set of 400 proteins as benchmark. The three methods are compared assuming an oracle predictor for the identification of CCD segments (i.e. we classify real CCD segments into the four oligomerization classes). In Table 5, we report a comparison of the three approaches in terms of per-class MCCs.

**Table 5.** CoCoNat and the state-of-art methods on the prediction of oligomerization states.

**Method** ^a^	MCC (parallel dimer)	MCC (antiparallel dimer)	MCC (trimer)	MCC (tetramer)
LOGICOIL	0.12	0.07	0.01	0.01
CoCoPRED	0.37	0.21	0.14	0.18
CoCoNat	**0.66**	**0.70**	**0.50**	**0.46**

a LOGICOIL (Vincent *et al.* 2013); CoCoPRED (Feng *et al.* 2022). The blind test set contains 400 positive proteins. Bold values highlight the highest scores.

Looking at the MCC values, CoCoNat significantly overpasses CoCoPRED and LOGICOIL, providing predictions that are overall higher and more balanced across the four oligomerization state classes. Remarkably, CoCoNat outperforms other tools also on less abundant classes, i.e. trimers and tetramers.

### 3.6 CoCoNat availability and analysis of running time

We release CoCoNat as both web server and standalone version. The web server is available at https://coconat.biocomp.unibo.it. The server provides a user-friendly web interface, allowing the user to choose between two modes of use of the tool: (i) analysis and visualization of coiled-coil prediction results for a single input sequence; (ii) submission of a batch job allowing prediction of coiled-coils and download of results (in TSV and JSON formats) for up to 500 sequences per job. Additionally, for single-sequence mode, we also provide the possibility of uploading a pre-determined set of coiled-coil segments, restricting the prediction to the oligomeric state only. The web application is implemented using Django (version 4.0.4), Bootstrap (version 5.3.0), JQuery (version 3.6.0), and neXtProt FeatureViewer (version 1.3.0-beta6) for visualization of predicted coiled-coil segments along the sequence.

The standalone version of CoCoNat is available on GitHub at https://github.com/BolognaBiocomp/coconat. The standalone tool is implemented in Python as a Docker containerized application. This avoids the installation of dependencies and allows users to quickly install the program in any server equipped with Docker. Instructions on how to build the Docker image and run CoCoNat are available on the GitHub repository.

We performed experiments to evaluate the running time of CoCoNat in different conditions. All the experiments were performed on the virtual machine hosting the web server, equipped with AMD EPYC 7301 12-Core Processor, 48G RAM. No GPU is available on this machine.

First, we analyzed the impact of the protein sequence length on the running time. To this aim, we randomly selected different sets of 100 sequences with lengths of increasing size. Fifty samples are generated for each length bin. CoCoNat has been then executed on each sample, evaluating its running time. Results are shown in Supplementary Fig. 3A. The running time scales linearly with the length of the sequences, ranging from 200 s (for 100 sequences) when protein length is 50–100 residues to 1400 s when length is 600/700 residues.

Second, we analyzed how the number of sequences in the dataset impacts on the running time. Again, random samples of sequences were generated, varying from 10, 20, 40, 80, 160, 320, and 500. The length of sequences was set between 100 and 200 residues for all samples. Results are reported in Supplementary Fig. 3B. The time required for the datasets including 10, 20, and 40 sequences is almost identical. This is due to the overhead required to load the two pLMs for encoding, which dominates the overall running time when the number of sequences is low. For dataset sizes including more than 40 sequences, the running time scales almost linearly from 100 to 1400 s.

In general, the CoCoNat running time is always low if compared to the time required by other tools based on multiple-sequence alignment inputs, such as CoCoPRED. For instance, to predict coiled-coil helices, including registers and oligomeric state, on 100 sequences of length comprised between 100 and 200 residues, the CoCoNat average running time is 330 s (5.5 min), which is lower than the time required by CoCoPRED, requiring about 2.5 h.

## 4 Conclusion

In this article, we described CoCoNat, a novel method based on protein language model embeddings and deep learning for detection of coiled-coiled helices at residue level, prediction of coiled-coil heptad repeat registers, and oligomerization state.

Training and testing were performed on datasets derived from literature. When compared with other state-of-the-art tools, CoCoNat reports performance that are significantly better than those obtained by other approaches tested, in particular when considering register and oligomerization state prediction.

In this work, we also proved the relevance of adopting protein residue representations derived from large-scale protein language models such as ProtT5 (Elnaggar *et al.* 2021) and ESM2 (Lin *et al.* 2023) for this specific task. Moreover, we further confirmed that the combination of different language models provides better performance, suggesting that different models obtained with different architectures and data give complementary representations.

## Supplementary Material

btad495_Supplementary_Data

## Data Availability

The data underlying this article are available in the article, in its online supplementary material and on the CoCoNat web server at https://coconat.biocomp.unibo.it.
