# Supplementary material for: CoCoNat: a novel method based on deep learning for coiled-coil prediction
Source: Bioinformatics. 2023 Aug 4;39(8):btad495. doi: 10.1093/bioinformatics/btad495 (PMC10425188; doi:10.1093/bioinformatics/btad495)
Supplement: btad495_Supplementary_Data [file btad495_supplementary_data.pdf]

Supplementary material

CoCoNat: a novel method based on deep-learning for coiled-coil prediction

Giovanni Madeo[1,°], Castrense Savojardo[1,°], Matteo Manfredi[1], Pier Luigi Martelli[1,*] and Rita Casadio[1]

[1]Biocomputing Group, Department of Pharmacy and Biotechnology, University of Bologna, Italy

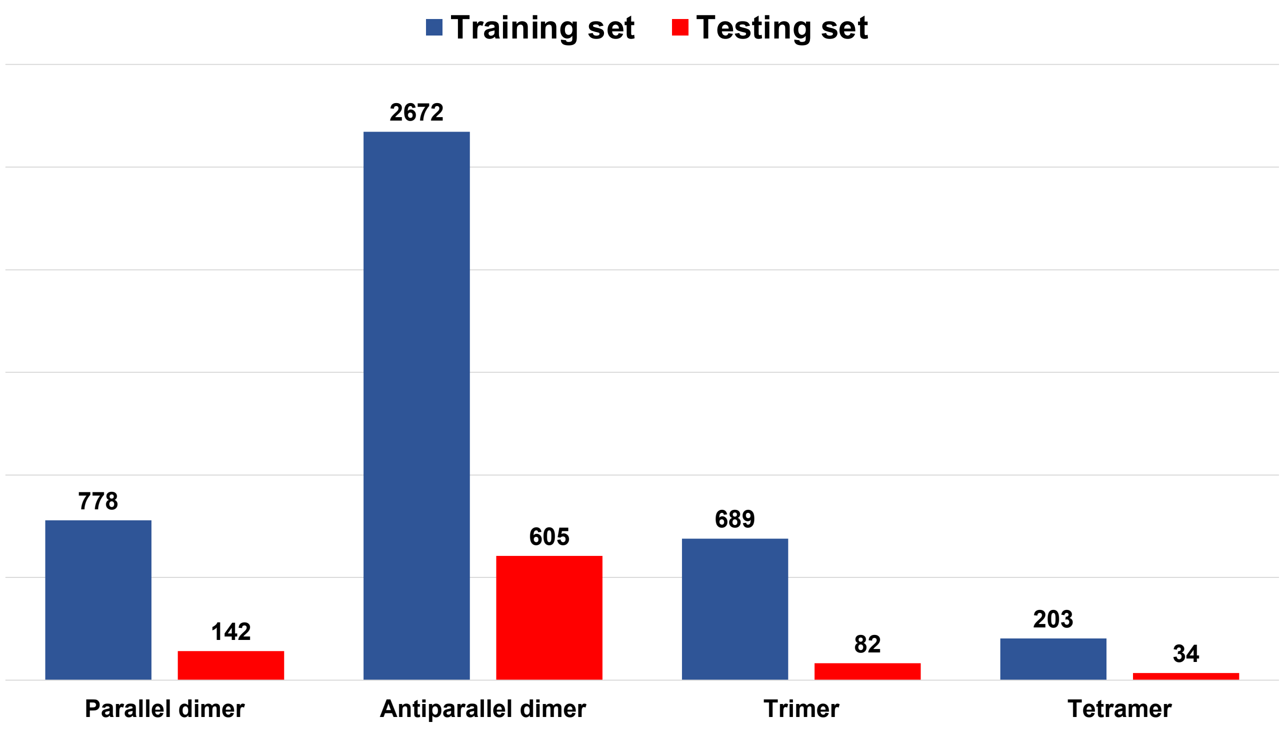

Supplementary Figure 1. Distribution of the four oligomeric states of coiled-coil helices in the training (blue) and testing (red) sets.

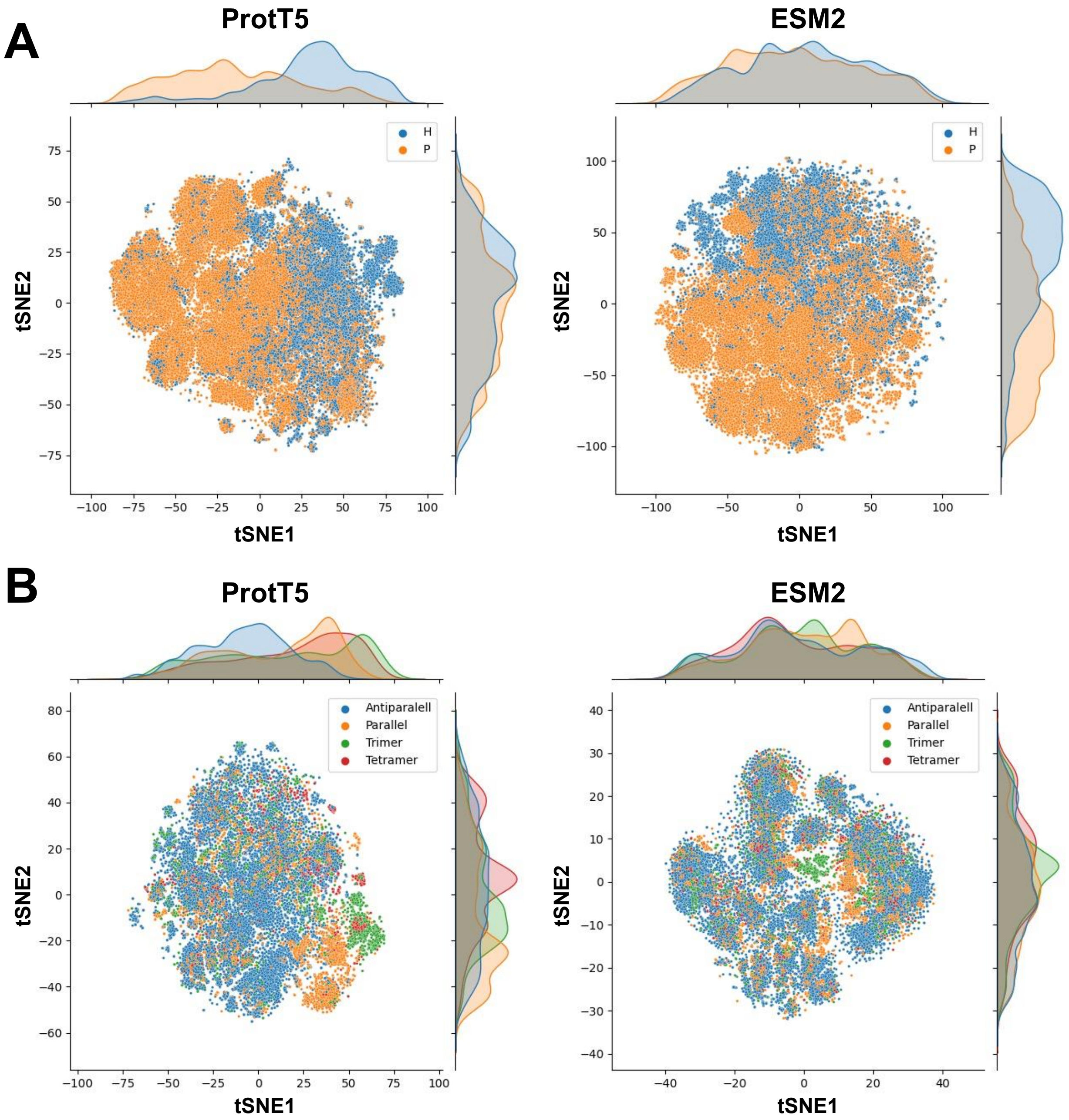

Supplementary Figure 2. t-SNE projections in two dimensions of raw embedding vectors obtained with ProtT5 and ESM2. **A:** Projections of representations of all coiled-coil residues in the training set. Colors of projected points reflect the heptad repeat register each residue is annotated with, distinguishing two classes: hydrophobic register positions (a and d, in blue) and polar positions (b, c, e, f and g, in orange). **B:** Projections of representations of residues annotated with hydrophobic registers (a and d). Colors of projected residue points reflect the oligomeric state of the corresponding helix.

Supplementary Table 1. Statistical significance (Welch's *t*-test) of the difference observed between CoCoNat and other tools in the prediction of coiled-coil helices.

**Method***	**PRER (p-value)**	**RECR (p-value)**	**F1R (p-value)**	**PRAUC (p-value)**	**PRES (p-value)**	**RECS (p-value)**	**F1s (p-value)**	**SOVO (p-value)**	**SOVP (p-value)**
**MarCoil**	0.35 (<0.0001)	0.26 (<0.0001)	0.29 (<0.0001)	0.17 (<0.0001)	0.24 (<0.0001)	0.06 (<0.0001)	0.10 (<0.0001)	18.05 (<0.0001)	48.64 (<0.0001)
**CCHMM_prof**	0.15 (<0.0001)	0.59 (<0.0001)	0.22 (<0.0001)	-	0.12 (<0.0001)	0.24 (<0.0001)	0.16 (<0.0001)	43.08 (<0.0001)	15.97 (<0.0001)
**MultiCoil2**	0.35 (<0.0001)	0.13 (<0.0001)	0.19 (<0.0001)	0.15 (<0.0001)	0.20 (<0.0001)	0.01 (<0.0001)	0.02 (<0.0001)	7.28 (<0.0001)	50.20 (<0.0001)
**DeepCoil2 (th=0.2)**	0.40 (<0.0001)	0.60 (<0.0001)	0.48 (<0.0001)	0.37 (<0.0001)	0.42 (<0.0001)	0.48 (<0.0001)	0.44 (<0.0001)	59.37 (<0.0001)	51.21 (<0.0001)
**DeepCoil2 (th=0.5)**	0.51 (<0.0001)	0.33 (<0.0001)	0.40 (<0.0001)	0.37 (<0.0001)	0.53 (<0.0001)	0.19 (<0.0001)	0.28 (<0.0001)	31.12 (<0.0001)	67.27 (0.98)
**CoCoPRED**	0.43 (<0.0001)	0.53 (0.18)	0.48 (<0.0001)	0.44 (<0.0001)	0.38 (<0.0001)	0.46 (<0.0001)	0.41 (<0.0001)	57.42 (<0.0001)	51.59 (<0.0001)
**CoCoNat**	0.55	0.53	0.54	0.47	0.56	0.43	0.48	54.28	67.28

MarCoil (Delorenzi and Speed, 2002); CCHMM_prof (Bartoli *et al.*, 2009); MultiCoil2 (Trigg *et al.*, 2011); DeepCoil2 (Ludwiczak *et al.*, 2019); CoCoPRED (Feng *et al.*, 2022). Subscript R: per residue; subscript S: per CC helix segment.

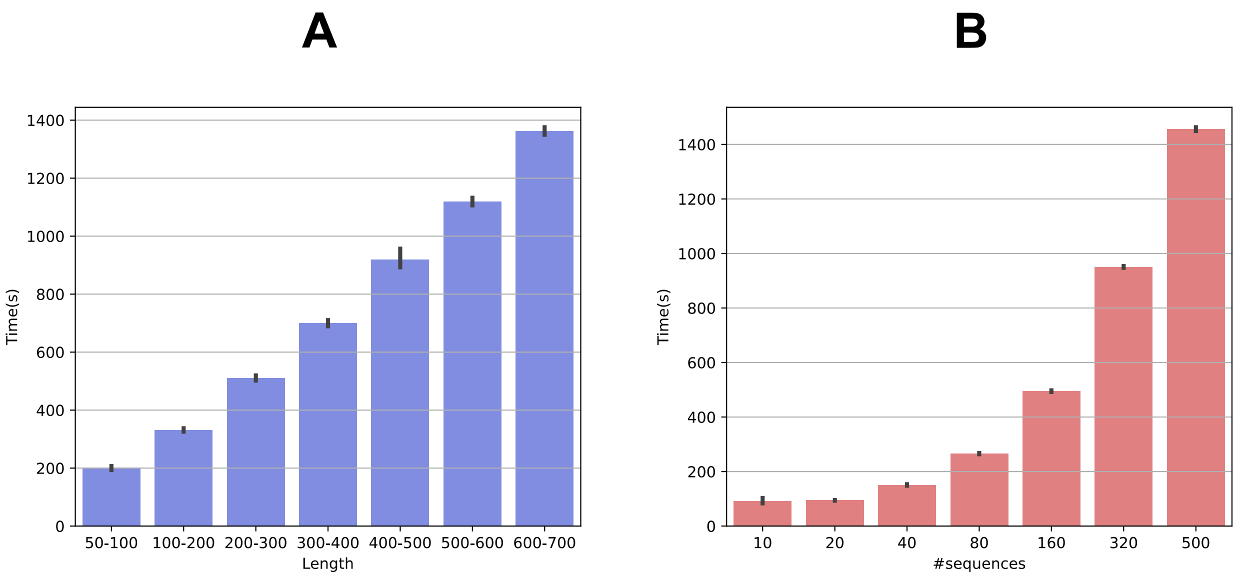

Supplementary Figure 3. CoCoNat running time analysis. **A**: the average running time is plotted as a function of the sequence length range. For each sequence length bin, 50 random samples including 100 random sequences are selected from UniProt. Reported time refer then to the processing of 100 sequences. **B**: the average running time, in seconds, as a function of the input dataset size. For each dataset size, 50 random samples including sequences of length 100-200 residue are selected from UniProt. All the experiments are executed on a virtual machine equipped with AMD EPYC 7301 12-Core Processor, 48G RAM (no GPU).
